# Impact of cancer-related and primary lymphedema and compression bandaging on limb range of motion: a cross-sectional study

**DOI:** 10.1007/s00520-026-10454-y

**Published:** 2026-03-11

**Authors:** Sara Bernasconi, Lorenzo Formichi, Giovanni Farina, Andrea Aliverti, Antonella LoMauro

**Affiliations:** 1 Dipartimento Di Elettronica, Informazione E Bioingegneria, Politecnico Di Milano, Piazza L. Da Vinci, 20133 Milan, Italy; 2 Istituti Clinici Zucchi Di Monza, Monza, Italy

**Keywords:** Lymphedema, Range of motion, articular, Bandages, Compression garment, Motion analysis system

## Abstract

**Introduction:**

Lymphedema is chronic and can be a consequence of cancer treatment. Little is known about the range of movement (ROM) of the limbs with lymphedema. We aimed to quantify the ROM in patients with lymphedema to assess the impact of lymphedema and multilayer bandaging on mobility.

**Methods:**

A motion analysis system quantified ROM. The ankle and knee of 22 patients (57 years, 14 females) with lower limb lymphedema (mainly secondary to gynecological or urological cancer) were evaluated. The wrist and elbow of 21 women (58 years) affected by upper limb lymphedema secondary to breast cancer were studied. Tests were repeated on the lymphedematous limb before (L) and after (B) bandaging, and with the compression garment (G, only for upper limb). The contralateral healthy limb (H) was set as a reference.

**Results:**

Lymphedema limited the knee maximal flexion (H 97.7°; L 83.1°) and the forearm rotation (H 140°, L 131°). Bandaging further limited the maximal knee ROM (70°). Bandaging restricted the maximal ROM of the ankle, elbow (H 147°, B 130°; only flexion limited), and wrist (H 113°, B 86°; both extension and flexion limited). Bandaging limits the ROM of the knee (H 40.7°; B 36.6°), ankle (H 29.6°; B 25.3°), and elbow (L 59°, B 54°) during the dynamic test. G limited the rotation of the forearm (111°). Data reported as median.

**Conclusion:**

Lymphedema and its treatment introduce important restrictions on joint mobility that may impact the quality of life, as adequate joint dorsiflexion is necessary for daily functional activities.

**Supplementary Information:**

The online version contains supplementary material available at 10.1007/s00520-026-10454-y.

## Introduction

Lymphedema is a chronic condition caused by impaired lymphatic drainage, leading to fluid accumulation in the interstitial space, swelling, and functional limitations in the affected limbs. Lymphedema can result in pain, skin changes, increased susceptibility to infections, and progressive fibrosis if left untreated [1–4]. The World Health Organization estimates a global prevalence of approximately 300 million cases [5–7], making it a significant public health concern. Lymphedema can be classified into two main categories: primary and secondary lymphedema [8]. The former arises from congenital abnormalities or genetic mutations affecting the development of lymphatic vessels. The latter is more common and secondary to oncologic treatments such as radiotherapy and surgical lymph node dissection. Currently, there is no definitive cure for lymphedema, but several therapeutic approaches are available to manage its symptoms and prevent disease progression [9–12]. The gold standard for treatment is complex decongestive therapy. Complex decongestive therapy combines manual lymphatic drainage, compression therapy, skin care, and physical exercise to reduce swelling and improve lymphatic function. Compression therapy plays a crucial role in fluid management by applying external pressure to facilitate lymphatic flow [12–17]. The primary objective of compression bandaging is to facilitate lymphatic return and reduce fluid accumulation through external pressure. However, compression bandaging inevitably imposes mechanical constraints on joint mobility and soft tissue flexibility [18]. The impact of compression therapy on joint mobility remains an area of debate. Excessive compression may restrict movement, while insufficient compression may fail to control swelling effectively. The swelling induced by lymphedema can lead to mechanical impairment in the limb’s movement. The range of motion (ROM) of the affected limb can therefore be potentially restricted [19]. Although it is commonly assumed that lymphedema may limit joint mobility due to tissue fibrosis and increased limb volume, direct scientific evidence supporting this claim is still limited. Previous research paid attention to the scapular girdle, finding a restricted shoulder ROM [20–24]. Very few studies in the literature investigated the elbow and the wrist with conflicting results, reporting a smaller ROM [25] or no deficit [26].

Upper extremity function was assessed via subjective (i.e., visual analogue scales and the Disabilities of the Arm, Shoulder and Hand questionnaire[27]) but not using objective techniques.

The knee was only investigated in case reports [28, 29] or during video recordings [30], while the ROM of the ankle received no attention. There is an important lack of knowledge in the pathophysiology of lymphedema, including understanding if and how the condition, the multilayer bandaging, and the compression garment [31] affect the ROM of the joints involved.

This study aimed to quantify the ROM in patients with lymphedema and to assess the impact of multilayer bandaging on the mobility in both limbs. This analysis could play an important role by quantifying the impairment caused by the condition and by the multilayer compression therapy to provide essential insights to tailor rehabilitation strategies.

## Materials and methods

The study was a cross-sectional prospective study. The research protocol of this study was approved by the local research Ethics Committee of Politecnico di Milano (approving number 20/2024) according to the Declaration of Helsinki. All participants signed a written informed consent form approved by the Data Protection Officer of Politecnico di Milano.

Patients were selected according to the following inclusion criteria: (1) confirmed diagnosis of lymphedema; (2) age > 18 years at the time of enrollment; (3) stable lymphedema stage (stages I to III) for at least 3 months before participation, based on the International Society of Lymphology (ISL) classification; (4) ability to ambulate independently without assistive devices; (5) no history of acute infection in the affected limb; (6) no history of previous orthopedic injury or musculoskeletal impairment in either the affected or unaffected limb; (7) willingness and ability to comply with study procedures; and (8) written informed consent obtained prior to participation.

Patients who did not meet all seven criteria of inclusion were not enrolled for the study.

### ROM assessment

**Fig. 1:**
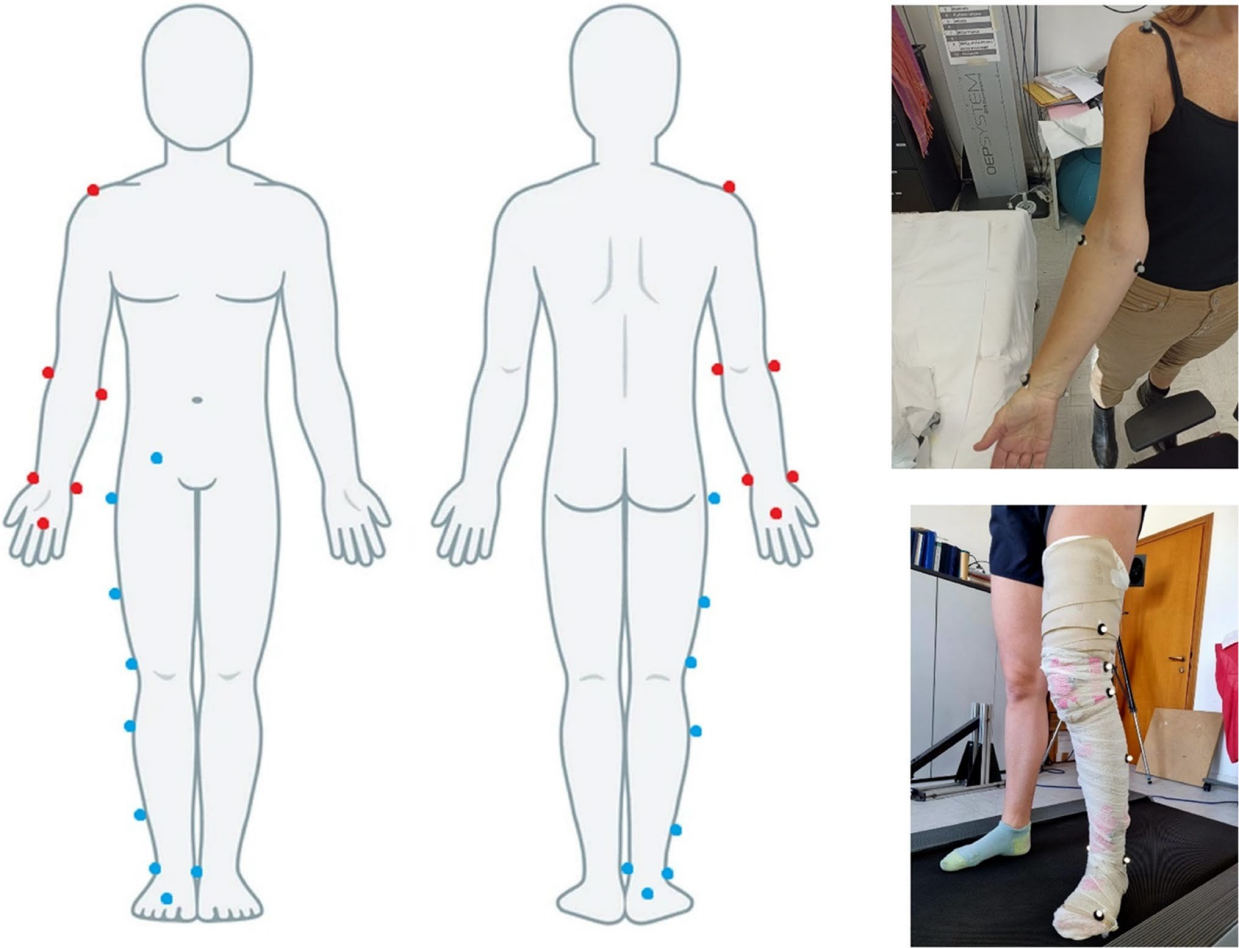
Protocol of marker placement protocols for the lower (blue points) and upper (red points) limb

This study was conducted using a motion analysis system (SMART system, BTS Bioengineering, Milan, Italy) based on an optoelectronic stereophotogrammetric approach with eight cameras recording at 100 Hz. Passive spherical markers were placed on the studied limb. For the lower limb, the Davis protocol was chosen with markers positioned on the anterior superior iliac spine, great trochanter, one-third of the leg, malleoli, metatarsal head of the middle finger, and heel. Finally, three markers were placed near the knee (Fig. 1). For the upper limb, markers were placed at the following anatomical landmarks: one on the acromion, two on the medial and lateral elbow, two on the wrist corresponding to the radius and ulna, and one on the metacarpal of the middle finger of the hand [32]. An additional marker was placed in the same position on the palm (Fig. 1). Acquisition protocols were performed at Politecnico di Milano under the supervision of a physiotherapist with 20 years of experience in lymphedema treatment who handled all the bandaging procedures. The lower limb protocol was previously described, and it comprised the following: (1) maximal flexion–extension of the ankle; (2) maximal flexion–extension of the knee; and (3) 1-min treadmill walking at 3 km/h (an affordable velocity for all the patients according to their physical condition). Each movement was repeated five or six times. The patient was in an orthostatic position, and the tests were repeated three times: on the lymphedematous lower limb before bandaging (L), on the lymphedematous lower limb after bandaging (B), and the contralateral healthy limb (H) set as a reference [33].

The upper limb protocol comprised (1) maximal flexion–extension of the elbow; (2) maximal flexion–extension of the wrist; (3) maximal pronation–supination of the forearm; and (4) 1-min tabletop cycling at controlled cadence (50 BPM).

Tests 1, 2, and 3 were performed with the patients in the supine position on a hospital bed. To focus on the specific movement and to minimize compensatory actions from other anatomical regions, the arm of interest was positioned with the elbow at the edge of the bed and the forearm hanging off it. The relative angle between the trunk and the arm was set at 45°. The starting position of the hand was supine for the flexion–extension of the elbow, pronated for the flexion–extension of the wrist and neutral for the pronation–supination of the forearm. During the pronation–supination of the forearm, the physiotherapist stabilized the arm of the patient with his hand to isolate the movement of the forearm and avoid arm rotation. Each movement was repeated five or six times. A tabletop bicycle was used for the dynamic trial. The tabletop bicycle was on the bed, and the patients were seated in front of the bed, adjusted to navel height. To standardize the bicycle distance, the starting position was set with one arm fully extended and the pedals horizontal. A metronome (50 BPM) controlled the execution speed, with the patient performing a half-turn per beat, alternating between the healthy and lymphedematous arms. The tests were repeated four times: on the lymphedematous upper limb before bandaging (L), on the lymphedematous upper limb after bandaging (B), on the lymphedematous upper limb with the patient’s compression garment (G), and the contralateral healthy limb (H) set as a reference.

### Lymphedema severity classification

The severity of lymphedema was assessed according to the International Society of Lymphology (ISL), and it was classified as mild (5–20% increase in limb volume), moderate (20–40%), or severe (> 40%) [9]. The volume differences were determined using the circumferential measurement, previously described and validated [34]. The circumferences were measured by the same operator following a segmental proportional technique: i.e., the limb length was fractionated at standardized distances following a constant proportion. The global limb volume was then calculated using the truncated cone formula [9, 16, 34].

### Bandaging procedure

The bandaging was performed by the same physiotherapist, who has 20 years of experience in lymphedema treatment and who supervised the acquisitions. The bandaging procedure consisted of a multilayer and multi-component system (Lohmann & Rauscher), which involves the use of the following materials: inelastic tubular bandage (TG7-TG9), German cotton, cohesive interposition bandage (this bandage had a maximum extensibility of 85% of its resting length; during the tests, the extensibility was limited to only 44.7% through a specific elliptical marker), short-stretch bandage (this bandage has a maximum extensibility of 49% of its resting length; during the tests, the extensibility was limited to only 50% through a specific elliptical marker), and cohesive fixation bandage with a protective (and not compressive) function [35, 36]. The bandage was printed with oval pressure indicators that became circles when the correct bandage stretch was achieved, and a consistent 50% overlap of the bandage was maintained on linear segments (i.e., the arm, the forearm, the leg, and the thigh). Standard procedures at the level of joint passages were used to try to guarantee the lowest restriction. In more detail, the bandaging was applied in a spiral manner, with the band orientation at 30° relative to the limb, each layer alternating in the opposite direction to the one underneath. The bandaging also included a series of additional steps near the knee, ankle, and elbow joints. Specifically, a transverse pass was performed first, followed by a descending and an ascending pass, before continuing with the spiral pattern, corresponding to a double overlap. For simplicity and uniformity of the tests, the bandaging performed involved a 50% overlap of the bandages along the entire limb [35, 36]. Similar procedures and materials were used for the upper limb. However, the hand bandaging was simplified for convenience: the short-stretch bandage was not applied in this anatomical region, but only a tightly woven cohesive bandage, as the fingers are not the focus of this study.

### Data analysis

SMARTAnalyzer (BTS Bioengineering, Milan, Italy) and a dedicated software developed on MATLAB computed the measurements of the angles recorded instant by instant for all the considered joints. The joint angles were calculated as follows: (1) the knee angle as the angle between the segments connecting the joint center of the knee with the two adjacent markers; (2) the ankle angle as the angle between the segment that connects the tip of the foot to the medial ankle and the segment that connects the medial ankle to the most distal marker placed on the leg; (3) the elbow angle as the angle between the segments connecting the midpoint of the elbow with the midpoint of the wrist and the acromion; (4) the wrist angle as the angle between the segments connecting the marker positioned on the metacarpal of the middle finger with the midpoints of the wrist and elbow; (5) the angle during the forearm rotation as the Euler angle between two reference systems placed at the midpoints of the elbow and wrist, respectively.

The ROM of the joints during maximal movements was computed as the difference between the minimum and the maximum angle reached during the five to six maneuvers.

The ROM of the joints during dynamic assessments was calculated based on the normalized step for the lower limb and the normalized pedal revolution for the upper limb.

### Statistical analysis and power computation

Statistical analysis was performed using RStudio. Data distribution was assessed using the Shapiro–Wilk test, and normality was assumed if the *p*-value > 0.05. Comparisons among conditions (healthy limb, lymphedematous limb, bandaged limb, or compression garment) were performed using the Friedman test with Dunn post hoc analysis for non-normally distributed data. Comparisons between primary and secondary lymphedema were performed using the Wilcoxon-Mann-Whitney *U* test for non-normally distributed data (SigmaStat 3.5, Systat Software, San Jose, CA). A pilot study on five subjects affected by lower limb lymphedema was conducted using an IMU system (XSENS, Movella [37]) to measure the mean and the standard deviation of the knee ROM during maximal flexion–extension of the same lymphedematous limb with (65 ± 25°) and without (92 ± 39°) the multilayer bandaging. Considering a significance level of 0.05, a power of 0.90, and a two-tailed test, the resulting total sample size was 19 subjects (G*Power Version 3.1.9.4). We have considered the same sample size for the upper limb.

## Results

### Lower limb

Twenty-two patients (median age 55.5 years, 14 females) were prospectively analyzed: seven were affected by mild (i.e., 5–20% increase in limb volume compared to the contralateral healthy limb [9]), 12 by moderate (i.e., 20–40% increase in limb volume compared to the contralateral healthy limb [9]), and three by severe lower limb lymphedema (i.e., > 40% increase in limb volume compared to the contralateral healthy limb [9]). Seventeen cases of lymphedema were secondary to gynecologic and/or urologic and/or pelvic oncological treatment or surgery, while five cases were primary. Data on age, sex, surgical procedure, tumor, lymphedema characteristics, and compression garment specifications for cases involving the lower limbs are summarized in Table 1.

**Table 1:** Data regarding age, sex, surgical intervention, lymphedema characteristics, and compression garment specifications for the cases involving the lower limbs

Age (years)	Sex	Lymhedema			Compression garment			
		Type	Severity	Tumor	Realization	Type of knit	Compression class	Product
71	M	Secondary	Moderate	Prostatic	Custom-sized	Flat	III	Above-knee with silicon border
73	F	Secondary	Moderate	Uterine	Standard	Flat	II	Above-knee with silicon border
77	F	Secondary	Severe	Uterine	Standard	Flat	II	Above-knee with silicon border
53	F	Secondary	Mild	Uterine	Custom-sized	Flat	II	Above-knee with silicon border
35	M	Primary	Moderate	-	Standard	Flat	II	Above-knee with silicon border
52	M	Secondary	Mild	Nevus	Custom-sized	Flat	II	Above-knee with silicon border
49	F	Primary	Moderate	-	Standard	Flat	II	Above-knee with silicon border
59	F	Secondary	Moderate	Uterine	Custom-sized	Flat	III	Tights
52	F	Secondary	Moderate	Uterine	Custom-sized	Flat	III	Tights
39	F	Secondary	Mild	Rhabdomyosarcoma	Standard	Flat	II	Above-knee with silicon border
77	M	Secondary	Severe	Prostatic	Custom-sized	Flat	III	Above-knee with silicon border
62	F	Secondary	Moderate	Uterine	Custom-sized	Flat	II	Above-knee with silicon border
66	F	Secondary	Moderate	Uterine	Standard	Flat	II	Above-knee with silicon border
66	M	Secondary	Moderate	Prostatic	Standard	Flat	II	Above-knee with silicon border
57	F	Secondary	Moderate	Nevus	Custom-sized	Flat	II	Above-knee with silicon border
52	F	Secondary	Mild	Nevus	Standard	Flat	II	Above-knee with silicon border
43	F	Primary	Severe	-	Custom-sized	Flat	III	Above-knee with silicon border
25	F	Primary	Mild	-	Custom-sized	Flat	III	Above-knee with silicon border
44	F	Secondary	Mild	Uterine	Custom-sized	Flat	III	Tights
74	M	Secondary	Moderate	Prostatic	Custom-sized	Flat	III	Above-knee with silicon border
54	F	Primary	Mild	-	Custom-sized	Flat	II	Above-knee with silicon border
64	M	Secondary	Moderate	Penile	Custom-sized	Flat	II	Above-knee with silicon border

We found no differences according to the etiology (Online Resources 1 and 2 reported all data and *p*-values) as patients with primary and secondary lymphedema showed comparable clinical and functional behavior, with no statistically or clinically meaningful differences. This finding suggests that primary and secondary lymphedema can reasonably be considered a homogeneous population.

#### Knee

**Fig. 2:**
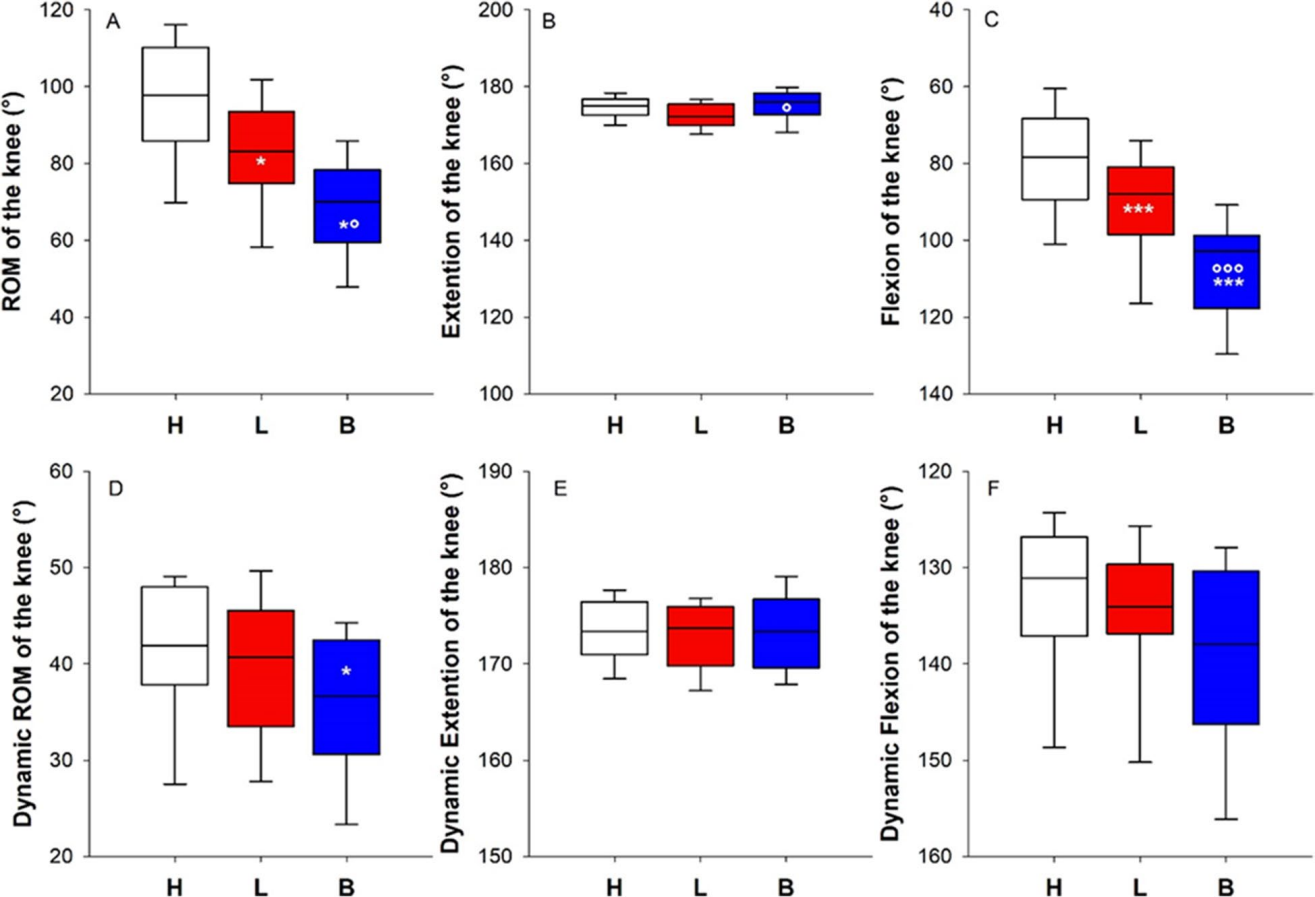
Box-and-whisker plot representing the median (line within the box), the interquartile range (length of the box), the 90th and the 10th percentiles (whiskers above and below the box) of the maximal ROM of the knee (**A**) and its two components: the extension (**B**) and the flexion (**C**) of the healthy limb (H, white); the lymphedematous limb (L, red) and the bandaged lymphedematous limb (B, blue). *^,^***p < 0.05, 0.001 vs H; °^,^°°°*p* < 0.05, 0.001 vs L. Box-and-whisker plot representing the median (line within the box), the interquartile range (length of the box), the 90th and the 10th percentiles (whiskers above and below the box) of the ROM of the knee (**D**) and its two components: the extension (**E**) and the flexion (**F**) during walking on the treadmill of the healthy limb (H, white); the lymphedematous limb (L, red) and the bandaged lymphedematous limb (B, blue). *p < 0.05 vs H

Lymphedema introduced a clear reduction of the ROM of the knee compared to the contralateral healthy knee (13.1% of median reduction). The application of multilayer bandaging further reduced the ROM compared to the lymphedematous knee (15.1% of median reduction). The knee limitation seemed to be completely attributed to the flexion movement. The multilayer bandaging limited the ROM of the knee also during walking, while lymphedema per se seemed not to significantly alter gait dynamics (Fig. 2).

#### Ankle

**Fig. 3:**
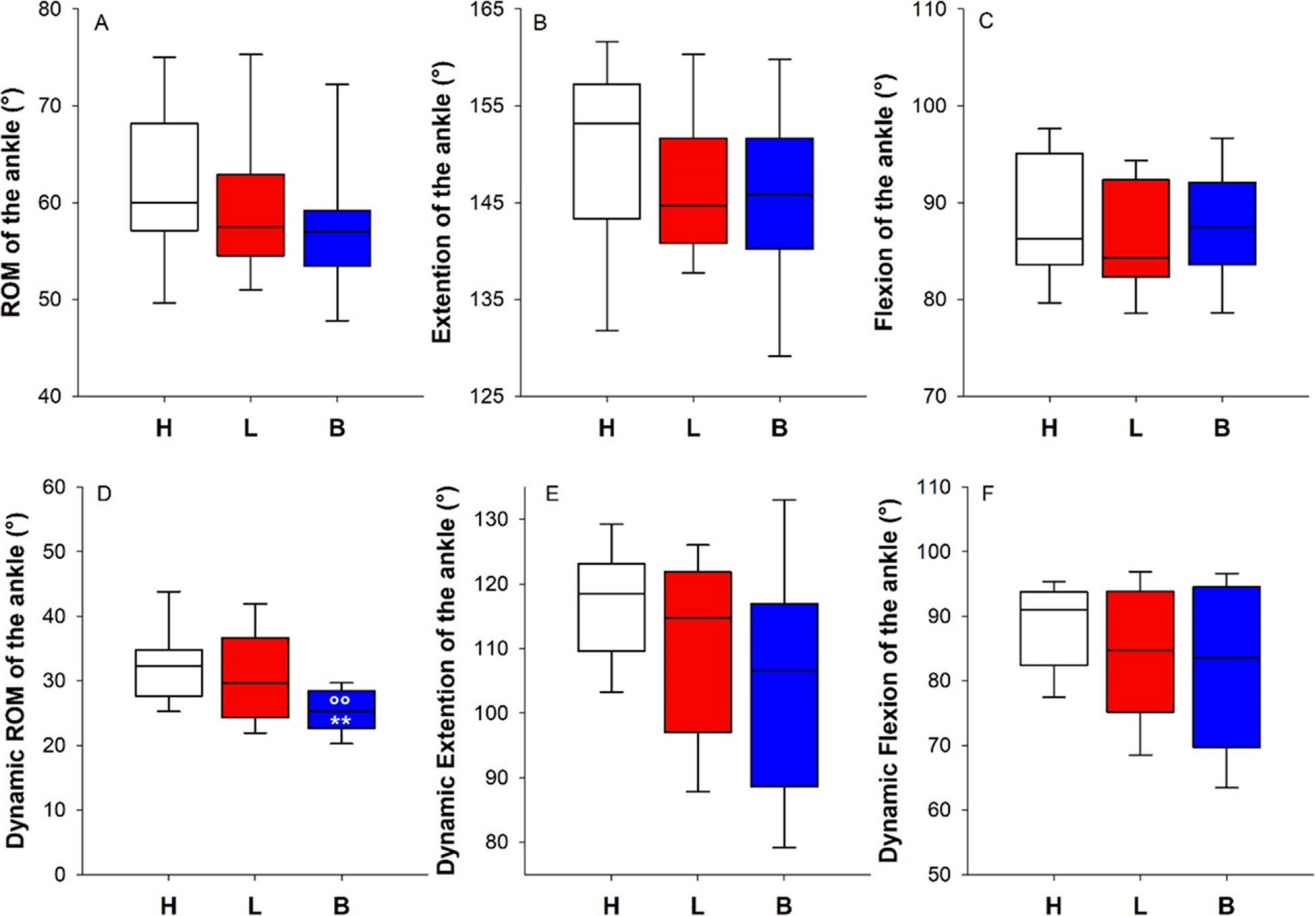
Box-and-whisker plot representing the median (line within the box), the interquartile range (length of the box), the 90th and the 10th percentiles (whiskers above and below the box) of the maximal ROM of the ankle (**A**) and its two components: the extension (**B**) and the flexion (**C**) of the healthy limb (H, white); the lymphedematous limb (L, red) and the bandaged lymphedematous limb (B, blue). Box-and-whisker plot representing the median (line within the box), the interquartile range (length of the box), the 90th and the 10th percentiles (whiskers above and below the box) of the ROM of the ankle **(D**) and its two components: the extension (E) and the flexion (**F**) during walking on the treadmill of the healthy limb (H, white); the lymphedematous limb (L, red), and the bandaged lymphedematous limb (B, blue). **p < 0.01 vs H; °°*p* < 0.01 vs L

 Neither the lymphedema nor the multilayer bandaging seemed to limit the maximal ROM of the ankle. However, the multilayer bandaging limited the ROM of the ankle during walking compared to both the healthy and the lymphedematous conditions, presumably due to the extension of the ankle. Indeed, the differences in the median values of the extension among the treatment groups were statistically significantly different (*p* = 0.034), but the multiple comparison procedure did not isolate the group or groups that differed from the others (Fig. 3).

### Upper limb

Twenty-one women (median age 58 years) were prospectively analyzed: four were affected by mild (i.e., 5–20% increase in limb volume compared to the contralateral healthy limb [9]), 11 by moderate (i.e., 20–40% increase in limb volume compared to the contralateral healthy limb [9]), and six by severe upper limb lymphedema (i.e., > 40% increase in limb volume compared to the contralateral healthy limb [9]). The lymphedema was secondary to breast cancer-related oncological treatment or surgery in all but one woman, whose lymphedema was secondary to radiotherapy for a lymphoma.

Data on age, sex, tumor, lymphedema characteristics, and compression garment specifications for cases involving the lower limbs are summarized in Table 2.

**Table 2:** Data regarding age, sex, tumour, lymphedema characteristics, and compression garment specifications for the cases involving the upper limbs

Age (years)	Sex	Lymhedema			Compression garment			
		Type	Severity	Tumor	Realization	Type of knit	Compression class	Product
72	F	Secondary	Moderate	Mammary	Standard	Circular	II	Glove + sleeve
65	F	Secondary	Moderate	Mammary	Standard	Flat	II	Glove + sleeve
57	F	Secondary	Mild	Mammary	Standard	Flat	II	Glove + sleeve
68	F	Secondary	Moderate	Mammary	Standard	Flat	II	Glove + sleeve
53	F	Secondary	Moderate	Lymphoma	Custom-sized	Flat	II	Sleeve
58	F	Secondary	Severe	Mammary	Standard	Flat	II	Glove + sleeve
62	F	Secondary	Severe	Mammary	Standard	Flat	II	Glove + sleeve
57	F	Secondary	Severe	Mammary	Standard	Flat	II	Glove + sleeve
53	F	Secondary	Moderate	Mammary	Standard	Flat	II	Glove + sleeve
67	F	Secondary	Mild	Mammary	Standard	Flat	II	Glove + sleeve
52	F	Secondary	Mild	Mammary	Standard	Flat	II	Glove + sleeve
77	F	Secondary	Moderate	Mammary	Standard	Flat	II	Glove + sleeve
61	F	Secondary	Severe	Mammary	Standard	Flat	II	Glove + sleeve
75	F	Secondary	Moderate	Mammary	Standard	Circular	II	Glove + sleeve
53	F	Secondary	Mild	Mammary	Standard	Flat	II	Glove + sleeve
51	F	Secondary	Moderate	Mammary	Standard	Flat	II	Glove + sleeve
46	F	Secondary	Severe	Mammary	Standard	Flat	II	Glove + sleeve
37	F	Secondary	Moderate	Mammary	Standard	Flat	II	Glove + sleeve
91	F	Secondary	Severe	Mammary	Standard	Flat	II	Glove + sleeve
45	F	Secondary	Moderate	Mammary	Standard	Flat	II	Glove + sleeve
70	F	Secondary	Moderate	Mammary	Standard	Flat	II	Glove + sleeve

#### Elbow

**Fig. 4:**
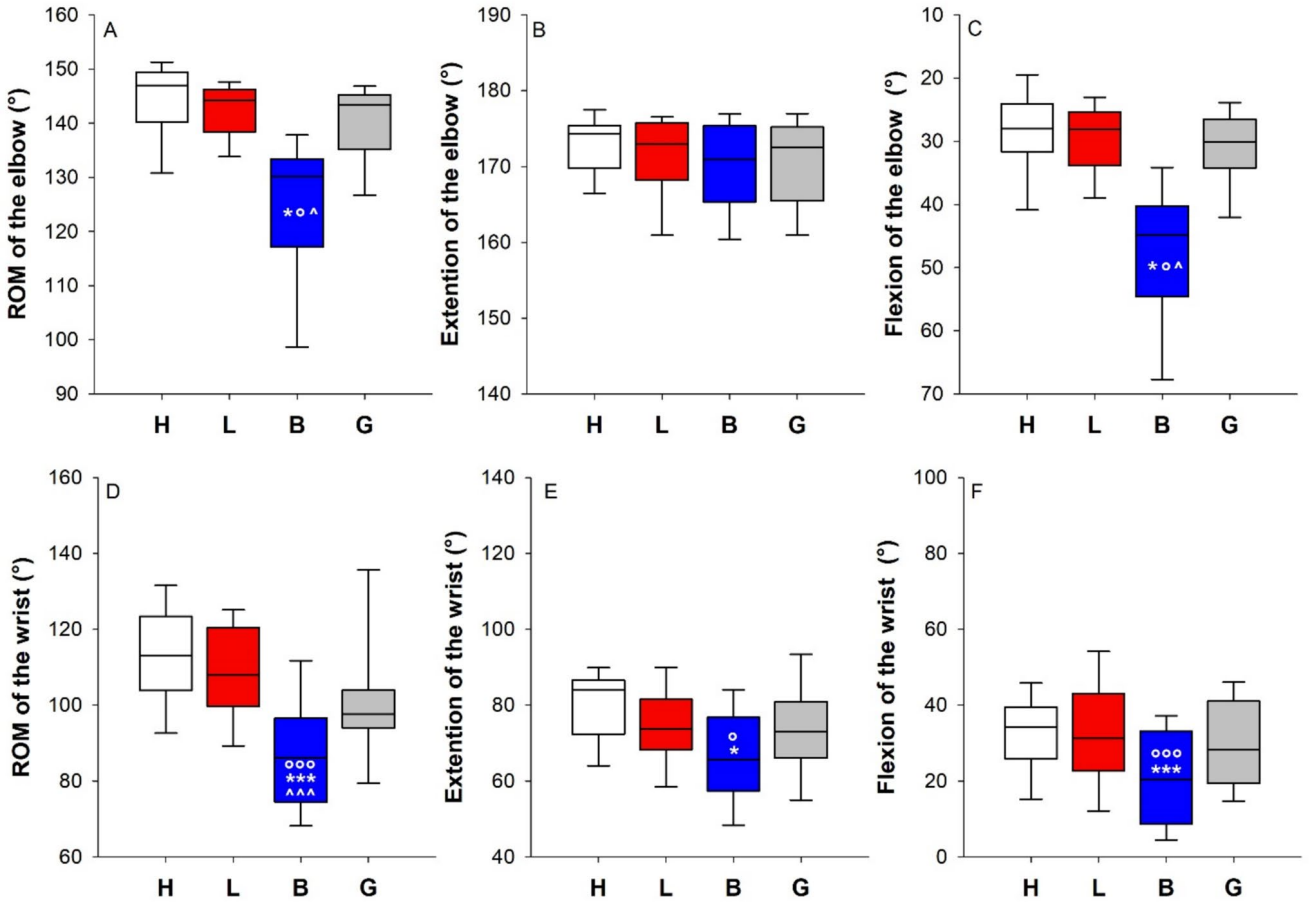
Box-and-whisker plot representing the median (line within the box), the interquartile range (length of the box), the 90th and the 10th percentiles (whiskers above and below the box) of the maximal ROM of the elbow (**A**) and its two components: the extension (**B**) and the flexion (**C**) of the healthy limb (H, white); the lymphedematous limb (L, red), the bandaged lymphedematous limb (B, blue), and the lymphedematous limb wearing the elastic garment (G, gray). *p < 0.05 vs H; °*p* < 0.05 vs L; ^*p* < 0.05 vs G. Box-and-whisker plot representing the median (line within the box), the interquartile range (length of the box), the 90th and the 10th percentiles (whiskers above and below the box) of the ROM of the wrist (**D**) and its two components: the extension (**E**) and the flexion (**F**) of the healthy limb (H, white); the lymphedematous limb (L, red), the bandaged lymphedematous limb (B, blue), and the lymphedematous limb wearing the elastic garment (G, gray). *^,^***p < 0.05, 0.001 vs H; °^,^°°°*p* < 0.05, 0.01 vs L; ^^,^^^^*p* < 0.05, 0.01 vs G

**Fig. 5:**
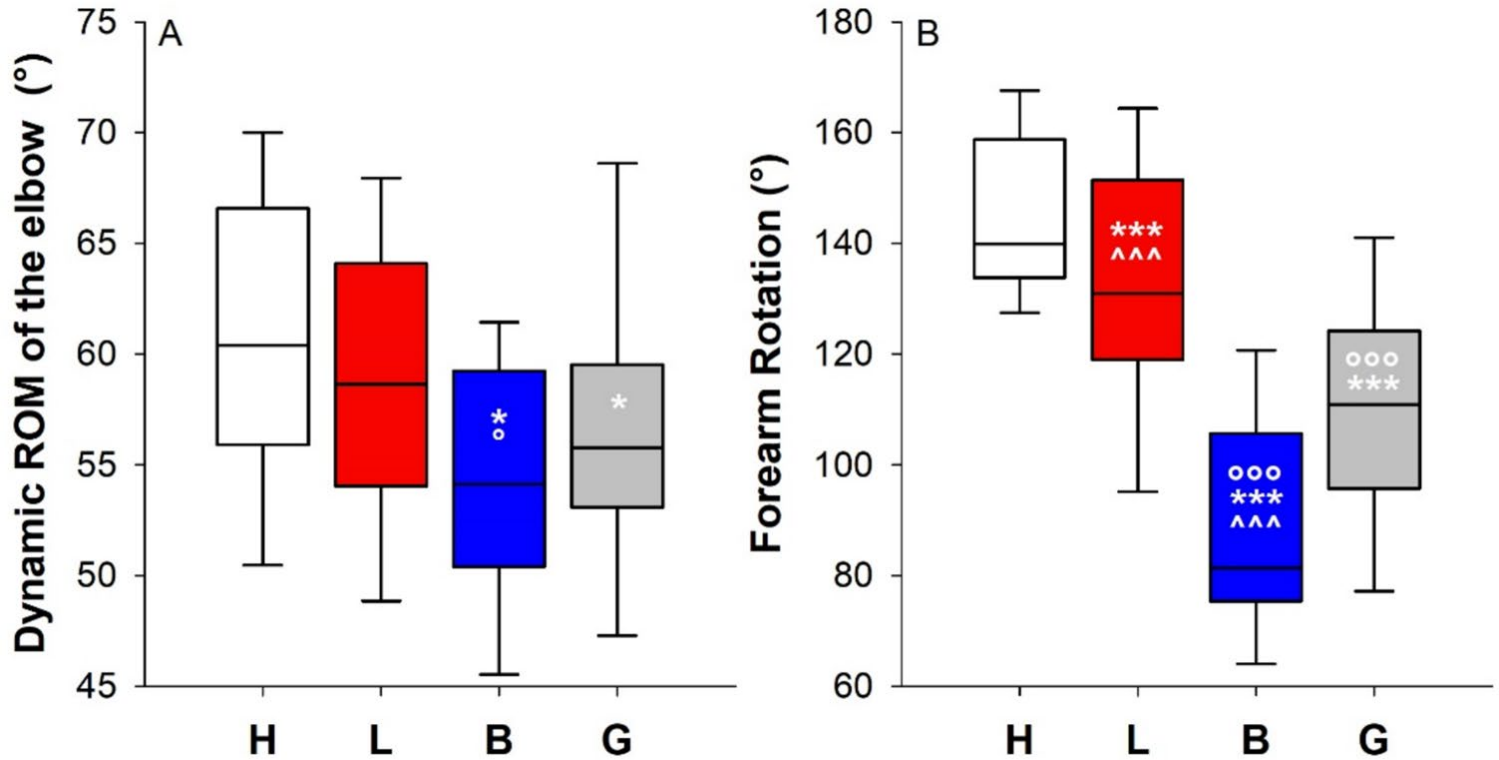
Box-and-whisker plot representing the median (line within the box), the interquartile range (length of the box), the 90th and the 10th percentiles (whiskers above and below the box) of the dynamic ROM of the elbow (**A**) of the healthy limb (H, white); the lymphedematous limb (L, red), the bandaged lymphedematous limb (B, blue), and the lymphedematous limb wearing the elastic garment (G, gray). **p < 0.01 vs H; °°*p* < 0.01 vs L. Box-and-whisker plot representing the median (line within the box), the interquartile range (length of the box), the 90th and the 10th percentiles (whiskers above and below the box) of the ROM of the pronation-supination of the forearm (**B**) of the healthy limb (H, white); the lymphedematous limb (L, red), the bandaged lymphedematous limb (B, blue), and the lymphedematous limb wearing the elastic garment (G, gray). ***p < 0.001 vs H; °°°*p* < 0.01 vs L; ^^^*p* < 0.001 vs G

The application of multilayer bandaging restricts the ROM of the elbow. The median ROM was 10.5% and 10% lower than that of the healthy and the lymphedematous limb, respectively. The restriction was entirely due to the flexion (Fig. 4, panels A, B, and C). Similar findings were observed during the dynamic analysis, with the multilayer bandaging restricting the elbow compared to the healthy and the lymphedematous limb. In addition, the elastic garment limits the elbow compared to the contralateral healthy limb (Fig. 5, panel A).

#### Wrist

The multilayer bandaging restricted the ROM of the wrist compared to the healthy limb, the lymphedematous limb, and the lymphedematous limb wearing the elastic garment. The restriction was due to both the extension and the flexion of the wrist (Fig. 4, panels D, E, and F).

#### Forearm rotation

The pronation-supination was the most affected movement, with all four conditions being significantly different from each other. The most restricted condition was the multilayer bandaging, followed by the elastic garment. Lymphedema, per se, limited the movement compared to the healthy contralateral limb (Fig. 5, panel B).

## Discussion

In this study, we have investigated the effect of lymphedema and multilayer bandaging on joint mobility of the upper and lower limbs. We have also evaluated the impact of the elastic garment on the upper limb. We have found that lymphedema, multilayer bandaging, and elastic garments introduced asymmetry compared to the contralateral healthy limb. Lymphedema alone limited the flexion of the knee, resulting in a restricted maximal ROM of the joint. Lymphedema per se also limited the rotation of the forearm. The multilayer bandaging further limited the maximal ROM of the knee. It also restricted the maximal ROM of the ankle, the elbow (because it limited the flexion), and the wrist (because it limited both the extension and the flexion). The multilayer bandaging had an important impact during the dynamic test of both limbs by limiting the ROM of the knee, the ankle, and the elbow. The rotation of the forearm seemed to be the most impacted movement because of lymphedema, multilayer bandaging, and the elastic garment limiting its mobility compared to the healthy contralateral forearm and among each other.

Our results have important implications for the pathophysiology and the treatment of lymphedema. Our research highlights the importance of understanding the impact of lymphedema and its treatment on joint mobility and, therefore, on the quality of life of these patients. Joint flexibility and mobility are components of the kinetic chain, the body’s coordination of various segments to perform specific activities involving precise positioning, timing, and speed. Any alteration in a component of the kinetic chain can develop compensatory patterns that may ultimately result in overuse and overload injuries [38]. Evaluating the ROM integrity in patients affected by lymphedema can significantly enhance efforts to improve their daily activities and mitigate resulting side effects. Adequate ankle and/or knee dorsiflexion is necessary for daily functional activities such as walking, jogging, and climbing stairs. Altered ROM of the ankle and/or the knee can put patients at risk of chronic biomechanical gait instability or be prone to injury or chronic pain [39]. We have shown that lymphedema per se implies asymmetric movements in the maximal flexion of the knee. We can speculate that this was due to the mechanical constraint of lymphedema that mainly accumulated around the knee. The multilayer bandaging further limited the maximal ROM of the knee, and it also impacted walking by limiting the ROM of the knee and the ankle.

A reduced motion capacity of the upper limb also has important implications. The upper limb function allows for complex task accomplishment in reaching, prehension, and manipulation, with the main effector being the hand. The wrist, elbow, and shoulder place the hand in space. We have shown that lymphedema per se limited the prono-supination of the forearm presumably because of the fibrotic tissue identified through the palpation of the skin. The multilayer bandaging strongly limited the maximal ROM of the elbow (because of flexion limitation), the wrist (because of extension limitation), and the forearm rotation. It also restricted elbow motion during the dynamic test. The compression garment limited the elbow during maximal and dynamic ROM and the rotation of the forearm.

For the first time, we have provided objective data on the functional status of the affected limb, showing movement limitations and asymmetries compared to the contralateral healthy limb. Asymmetric limb movement can lead to a range of biomechanical and functional issues, as compensatory mechanisms may develop. These compensations can result in abnormal loading of joints, muscle imbalances, and altered gait or posture [40–43]. Because lymphedema is a chronic condition, such adaptations may increase the risk of overuse injuries, joint degeneration, and chronic pain over time. Persistent asymmetry can hinder functional recovery, reduce the overall efficiency of the movement, and ultimately affect the individual’s quality of life and ability to perform daily activities. For these reasons, our attention to the ROM in joints affected by lymphedema is essential for several clinical and therapeutic reasons. A strength of our protocol was testing four different conditions. The comparison with the contralateral healthy limb allowed highlighting the presence of asymmetries that the patients must compensate for during their daily activities. The asymmetry found when the lymphedematous limb was bandaged provided insight into what happens to the patient during phase I of complex decongestive therapy. Complex decongestive therapy is recommended for patients to be treated at least once a year, five times per week, one session per day, for several weeks (typically 2 or 3). In the remaining days of the year and for all day, patients should wear elastic compression garments, which fit like a second skin, to maintain the swelling reduction achieved with complex decongestive therapy [1, 9, 11, 13]. The asymmetry found when the patients wore the elastic garment provided insight into what happens to the patient during the maintenance phase. The use of the elastic garment depends on the compliance of the patients [44, 45]. The asymmetry found when the lymphedematous limb was naked provided insight into what happens to the patient when they deliberately decide not to use the elastic garment. Therefore, the four conditions we analyzed cover all the different situations a lymphedematous limb experiences over time. Although we have tested a few movements, we have considered the body’s natural movement patterns, particularly for the lower limb (i.e., walking). Future studies should be addressed to study functional movements of the upper limb that mimic real-world activities and use of multiple joints and planes of motion, such as grasping, pointing, lifting, pushing, and pulling.

### Limitations of the study

Considering only the kinematics of the movement, without considering the forces and pressures involved and therefore the mechanics of the movement, was one limit of this study. Also, we have not computed the most important kinematic parameters (i.e., mean step length, gait line length, and cadence variability) during walking. This was a simple attempt to analyze walking in these patients, considering also that limited studies on the gait analysis in patients with lower limb lymphedema are available. As recently shown in a review article, only five articles considered walking and lower limb lymphedema. Of these, only two studies used gait analysis, while the other studies used videos and questionnaires [46]. Future studies should be dedicated to the gait analysis of these patients to better understand the biomechanics of walking in these patients, which could improve clinical knowledge and, consequently, the quality of life [46]. Our study represents an initial attempt to analyze gait in patients with lower limb lymphedema in relation to bandaging and is limited by its exploratory nature.

We deliberately kept the protocol of analysis simple to provide benchmarks to the clinicians. Although motion analysis systems are the gold standard for ROM assessment, they are not available to all clinicians due to their high cost, technical complexity, and the need for specialized training and equipment. Traditionally, in physical therapy, the ROM of a joint is measured by a goniometer, with each arm positioned at specific points on the body and the center of the goniometer aligned at the joint of interest [26]. Our protocol of marker positioning and data analysis, therefore, was close to this kind of measurement. Surrogate tools such as inertial measurement units are valuable because they provide accessible, cost-effective, and portable alternatives to traditional motion analysis systems. These surrogates would enable clinicians to quantitatively assess movement in a wider range of settings, including the compressive therapy [33].

Another limitation of the study was the low number of severe cases of lymphedema. Our results are therefore polarized towards milder forms. However, the lower incidence of severe lymphedema reflects the epidemiology of the condition. Indeed, early diagnosis, improved surgical techniques, immediate lymphatic reconstruction, increased awareness, and patient education contribute to earlier intervention, which can mitigate the progression to more advanced stages of lymphedema [47–49]. Future studies on larger populations should be aimed at stratifying the motion and the kinematics of lymphedematous limbs according to severity.

Being a single-operator study is an important limitation of our study. Our results on the effect of the compression bandaging cannot be considered universal because one of the primary challenges associated with lymphatic bandaging is its operator-dependent nature. The effectiveness of lymphatic bandaging relies heavily on the clinician’s skill and experience. The extent of permitted movement depends on factors such as the materials used, the type of bandage, the technique of application (i.e., layering, tensioning, and positioning of the bandages), and the level of compression exerted. An optimal bandaging technique should be a balance between providing adequate compression while allowing sufficient functional mobility to promote muscle pump activity, which is essential for effective lymphatic drainage. Excessive restriction may hinder active movement, reduce patient compliance, and negatively impact rehabilitation outcomes [27, 50, 51]. We have shown that bandaging can introduce important limitations to the joints during maximal movement and walking or dynamic rotation of the upper limb. Physiotherapists, therefore, must be aware of the movement restriction induced by their bandaging, as it can significantly influence both functional mobility and therapeutic outcomes. The subjectivity of the bandage technique limits standardization, and it may affect treatment outcomes and reproducibility across different practitioners. For this reason, future studies should be focused on evaluating different operators and different kinds of bandages [52]. However, we used a marking system on the bandage for tension control to help apply a consistent level of compression to reduce operator variability, therefore ensuring more consistent pressure application.

Another limitation of this study was that we evaluated only the acute effect (i.e., immediately after the application) of the bandage. It would be interesting to repeat the same evaluations (1) before bandage removal, to assess its effectiveness over time; (2) immediately after its removal, to verify whether ROM changes are already observable; and (3) at the end of the intensive treatment, after 10–15 days.

The last limitation was considering the compression garments only for the upper limb. However, these are the first results reported on the effect of compression bandaging in lymphedema. Understanding how compression garments work is crucial because they represent a cornerstone of the treatment during the maintenance phase of complex decongestive therapy that must be worn daily.

Although we also included five patients with primary lower limb lymphedema, this does not represent a limitation, as the etiology was shown not to be a discriminating factor [16, 53], and this was also confirmed in our set of data. Our analyses demonstrated that patients with primary and secondary lymphedema showed comparable clinical and functional behavior across the outcomes investigated. Our inclusive approach with primary lymphedema was meant to increase knowledge of this rare condition, which remains insufficiently explored in literature, and it is often underrepresented in clinical research.

## Conclusions

For the first time, the effects of lymphedema and its treatment on the ROM of the joints of both the lower and upper limbs were measured. The results highlight significant restricted effects of lymphedema, compression bandaging, and elastic garments on joint movement, with potential impact on patients’ ability to perform daily activities. Although these results applied only to the type of bandage that we used, we have shown that lymphatic bandaging directly influences the degree of movement permitted in the bandaged limb and introduces asymmetric movements. The subjectivity of the bandage technique limits standardization. Physiotherapists must be aware of the movement restriction induced by their bandaging. Understanding these biomechanical constraints is crucial for developing individualized rehabilitation plans to balance effective lymphedema management with the preservation of functional independence and quality of life. Regular ROM assessments can be crucial for designing individualized rehabilitation programs aimed at preserving or restoring joint function, preventing secondary complications such as contractures or musculoskeletal imbalances, and monitoring the effectiveness of therapeutic interventions over time and supporting evidence-based clinical decision-making.

## Supplementary Information

Below is the link to the electronic supplementary material.

ESM 1 (DOCX.485 KB)

## Data Availability

The data that support the findings of this study are available from the corresponding author, ALM, upon reasonable request. The corresponding author had full access to all of the data in this study and takes complete responsibility for the integrity of the data and the accuracy of the data analysis.

## References

[1] Wu T, Pu J, Yao Q et al (2025) Advances in etiology, pathophysiology, diagnosis, and management of lymphedema: a comprehensive review. Front Med. 10.3389/FMED.2025.1666522 41282012 10.3389/fmed.2025.1666522 PMC12631335

[2] Lee SO, Kim IK (2024) Molecular pathophysiology of secondary lymphedema. Front Cell Dev Biol. 10.3389/FCELL.2024.1363811 39045461 10.3389/fcell.2024.1363811 PMC11264244

[3] Azhar SH, Lim HY, Tan BK et al (2020) The unresolved pathophysiology of lymphedema. Front Physiol. 10.3389/FPHYS.2020.00137 32256375 10.3389/fphys.2020.00137 PMC7090140

[4] Grada AA, Phillips TJ (2017) Lymphedema: pathophysiology and clinical manifestations. J Am Acad Dermatol 77:1009–1020. 10.1016/j.jaad.2017.03.022 29132848 10.1016/j.jaad.2017.03.022

[5] Keast DH, Moffatt C, Janmohammad A (2019) Lymphedema impact and prevalence International Study: the Canadian data. Lymphat Res Biol 17:178–186. 10.1089/LRB.2019.0014 30995190 10.1089/lrb.2019.0014 PMC6639111

[6] McCall MK, Destin D, Miller ME et al (2025) Prevalence and predictive testing preferences for breast cancer treatment side effects. Support Care Cancer. 10.1007/S00520-025-09976-8 41261260 10.1007/s00520-025-09976-8 PMC12630186

[7] Gérard N, Farmakis IT, Valerio L et al (2025) Epidemiological study of lymphedema prevalence and comorbidities in hospitalized patients in the United States. J Clin Med. 10.3390/JCM14228156 41303192 10.3390/jcm14228156 PMC12653852

[8] Barone V, Borghini A, Tedone Clemente E et al (2020) New insights into the pathophysiology of primary and secondary lymphedema: histopathological studies on human lymphatic collecting vessels. Lymphat Res Biol 18:502–509. 10.1089/LRB.2020.0037 32716244 10.1089/lrb.2020.0037

[9] The diagnosis and treatment of peripheral lymphedema (2020) 2020 Consensus Document of the International Society of Lymphology. Lymphology 53:3–19. 10.2458/lymph.4649 32521126

[10] Mortimer PS. The pathophysiology of lymphedema. *Cancer* 1998;**Dec 15;83(**:2798–802. 10.1002/(sici)1097-0142(19981215)83:12b+<2798::aid-cncr28>3.3.co;2-5 9874400

[11] Hentati F, Donohoe K, Weinstein J et al (2025) Multidisciplinary approach to lymphedema diagnosis and management. Semin Vasc Surg. 10.1053/j.semvascsurg.2025.09.002 41386913 10.1053/j.semvascsurg.2025.09.002

[12] Yamamoto R, Yamamoto T (2007) Effectiveness of the treatment-phase of two-phase complex decongestive physiotherapy for the treatment of extremity lymphedema. Int J Clin Oncol 12:463–468. 10.1007/S10147-007-0715-5 18071866 10.1007/s10147-007-0715-5

[13] Chaker SC, James AJ, King D et al (2024) Lymphedema: current strategies for diagnostics and management. Ann Plast Surg. 10.1097/SAP.0000000000004044 39356288 10.1097/SAP.0000000000004044

[14] Mortimer PS, Rockson SG (2014) New developments in clinical aspects of lymphatic disease. J Clin Invest 124:915–921. 10.1172/JCI71608 24590276 10.1172/JCI71608 PMC3938261

[15] Martín Jiménez A, Ortega Nieto C, Lista S et al (2025) Effectiveness of the different components of complex decongestive therapy in patients with chronic venous insufficiency: a systematic review. Phlebology. 10.1177/02683555251333000 40180589 10.1177/02683555251333000

[16] Farina G, Santaniello I, Galli M et al (2025) Modeling of lymphedema distribution and complex decongestive therapy effectiveness. Lymphat Res Biol. 10.1177/15578585251387049 41054391 10.1177/15578585251387049

[17] Ko DSC, Lerner R, Klose G et al (1998) Effective treatment of lymphedema of the extremities. Arch Surg 133:452–458. 10.1001/ARCHSURG.133.4.452 9565129 10.1001/archsurg.133.4.452

[18] Gülören G, Doğan Y, Özgül S et al (2023) Acute effects of remedial exercises with and without compression on breast-cancer-related lymphedema. Healthcare. 10.3390/HEALTHCARE11222949 37998441 10.3390/healthcare11222949 PMC10671079

[19] Sleigh B, Manna B. *Lymphedema*. StatPearls [Internet]. 2023.

[20] Nesvold IL, Dahl AA, Løkkevik E et al (2008) Arm and shoulder morbidity in breast cancer patients after breast-conserving therapy versus mastectomy. Acta Oncol 47:835–842. 10.1080/02841860801961257 18568481 10.1080/02841860801961257

[21] Balzarini A, Lualdi P, Lucarini C, *et al.* Biomechanical evaluation of scapular girdle in patients with chronic arm lymphedema. *Lymphology* 2006;**Sep;39(3)**:132–40. 17036634

[22] Mohamed MH, Radwan RE, ElMeligie MM et al (2024) The impact of lymphedema severity on shoulder joint function and muscle activation patterns in breast cancer survivors: a cross-sectional study. Support Care Cancer. 10.1007/S00520-024-09044-7 39690278 10.1007/s00520-024-09044-7 PMC11652595

[23] Xu Q, Liu C, Jia S et al (2024) Effect of physical exercise on postoperative shoulder mobility and upper limb function in patients with breast cancer: a systematic review and meta-analysis. Gland Surg 13:1494–1510. 10.21037/GS-24-255/COIF) 39282024 10.21037/gs-24-255 PMC11399002

[24] Rezende MS, Rossi DM, de Ribeiro Lima AM et al (2024) Shoulder and scapulothoracic impairments in women with breast cancer-related lymphedema in the upper limb: a cross-sectional study shoulder and breast cancer-related lymphedema. J Bodyw Mov Ther 37:177–182. 10.1016/J.JBMT.2023.11.055 38432802 10.1016/j.jbmt.2023.11.055

[25] Haddad CAS, Saad M, Perez M del CJ, *et al.* Assessment of posture and joint movements of the upper limbs of patients after mastectomy and lymphadenectomy. *Einstein (Sao Paulo)* 2013;**11**:426–34. 10.1590/S1679-45082013000400004 10.1590/S1679-45082013000400004 PMC4880377 24488379

[26] Mazor M, Lee JQ, Peled A et al (2018) The effect of yoga on arm volume, strength, and range of motion in women at risk for breast cancer-related lymphedema. J Altern Complement Med 24:154–160. 10.1089/ACM.2017.0145 29064279 10.1089/acm.2017.0145

[27] King M, Deveaux A, White H et al (2012) Compression garments versus compression bandaging in decongestive lymphatic therapy for breast cancer-related lymphedema: a randomized controlled trial. Support Care Cancer 20:1031–1036. 10.1007/S00520-011-1178-9 21553314 10.1007/s00520-011-1178-9

[28] Holtgrefe KM (2006) Twice-weekly complete decongestive physical therapy in the management of secondary lymphedema of the lower extremities. Phys Ther 86:1128–1136. 10.1093/ptj/86.8.1128 16879046

[29] Sato F, Sakai H (2021) Improvement of knee joint’s range of motion in a patient with posterior thigh lymphedema by lymphaticovenular anastomosis. Microsurgery 41:816–817. 10.1002/MICR.30801 34462955 10.1002/micr.30801

[30] Aggithaya M, Narahari S, Ryan T (2015) Yoga for correction of lymphedema’s impairment of gait as an adjunct to lymphatic drainage: a pilot observational study. Int J Yoga 8:54. 10.4103/0973-6131.146063 25558134 10.4103/0973-6131.146063 PMC4278136

[31] Cheng H, Gong J, Yu L et al (2025) Compression sleeves for prevention and treatment of breast cancer-related lymphedema: a systematic review and meta-analysis. Breast Cancer Res Treat 215:41. 10.1007/S10549-025-07846-9 41389304 10.1007/s10549-025-07846-9

[32] Hebert JS, Lewicke J, Williams TR et al (2014) Normative data for modified box and blocks test measuring upper-limb function via motion capture. J Rehabil Res Dev 51:919–931. 10.1682/JRRD.2013.10.0228 10.1682/JRRD.2013.10.0228 25356979

[33] Bernasconi S, Oriolo G, Farina G et al (2025) Design and validation of a wearable system for enhanced monitoring of lower limb lymphedema. IEEE J Biomed Heal Informatics 13:193–201. 10.1109/JTEHM.2025.3563985 10.1109/JTEHM.2025.3563985 PMC12250963 40657523

[34] Farina G, Galli M, Borsari L et al (2024) Limb volume measurements: a comparison of circumferential techniques and optoelectronic systems against water displacement. Bioeng (Basel, Switzerland). 10.3390/BIOENGINEERING11040382 10.3390/bioengineering11040382 PMC11048605 38671803

[35] Farina G. Procedure algoritmiche applicate alla terapia compressiva: tecnica moderna di bendaggio linfologico. In: *Giornale Italiano di Linfologia G.I.L.* 2012.

[36] Farina G. L’uso dei sistemi di marcatura nel bendaggio multistrato. In: *Giornale Italiano di Linfologia G.I.L.* 2012.

[37] Rathi SG, Sharath H V, Kolhe PD. Effect of kinematic analysis on the gait of school-going children with different types of foot arches: an observational study using Xsens 3D motion technology. *Cureus* 2025;**17**. 10.7759/CUREUS.78322 10.7759/cureus.78322 PMC11873750 40034635

[38] Almansoof HS, Nuhmani S, Muaidi Q (2023) Role of kinetic chain in sports performance and injury risk: a narrative review. J Med Life 16:1591–1596. 10.25122/JML-2023-0087 38406779 10.25122/jml-2023-0087 PMC10893580

[39] Rao Y, Yang N, Gao T et al (2024) Effects of peak ankle dorsiflexion angle on lower extremity biomechanics and pelvic motion during walking and jogging. Front Neurol 14:1269061. 10.3389/FNEUR.2023.1269061 38362013 10.3389/fneur.2023.1269061 PMC10867967

[40] Mohamed A, Sexton A, Simonsen K et al (2019) Development of a mechanistic hypothesis linking compensatory biomechanics and stepping asymmetry during gait of transfemoral amputees. Appl Bionics Biomech. 10.1155/2019/4769242 30863460 10.1155/2019/4769242 PMC6378070

[41] Heil J, Loffing F, Büsch D (2020) The influence of exercise-induced fatigue on inter-limb asymmetries: a systematic review. Sports Med Open. 10.1186/S40798-020-00270-X 32844254 10.1186/s40798-020-00270-x PMC7447715

[42] Heil J (2022) Load-induced changes of inter-limb asymmetries in dynamic postural control in healthy subjects. Front Hum Neurosci. 10.3389/FNHUM.2022.824730 35360281 10.3389/fnhum.2022.824730 PMC8963187

[43] Wang P, Qin Z, Zhang M (2025) Association between pre-season lower limb interlimb asymmetry and non-contact lower limb injuries in elite male volleyball players. Sci Rep. 10.1038/S41598-025-98158-X 40281067 10.1038/s41598-025-98158-x PMC12032107

[44] Erdinç Gündüz N, Şahin E, Dilek B et al (2022) Adherence to compression garment wear and associated factors among patients with breast cancer-related lymphedema: a pilot study from a Turkish tertiary center. Lymphat Res Biol 20:665–670. 10.1089/LRB.2021.0091 35245100 10.1089/lrb.2021.0091

[45] Medina Rodríguez ME, Socorro Suárez R, Albornoz Cabello M et al (2025) Adherence to compression garments in lymphedema patients: a cross-sectional study. Medicina Kaunas. 10.3390/MEDICINA61040685 40282976 10.3390/medicina61040685 PMC12028548

[46] Tedeschi R (2023) Biomechanical alterations in lower limb lymphedema: implications for walking ability and rehabilitation. Phlebology 38:496–502. 10.1177/02683555231188236 37413662 10.1177/02683555231188236

[47] McLaughlin SA, Staley AC, Vicini F et al (2017) Considerations for clinicians in the diagnosis, prevention, and treatment of breast cancer-related lymphedema: recommendations from a Multidisciplinary Expert ASBrS panel: part 1: definitions, assessments, education, and future directions. Ann Surg Oncol 24:2818–2826. 10.1245/S10434-017-5982-4 28766232 10.1245/s10434-017-5982-4

[48] McLaughlin SA, DeSnyder SM, Klimberg S et al (2017) Considerations for clinicians in the diagnosis, prevention, and treatment of breast cancer-related lymphedema, recommendations from an expert panel: part 2: preventive and therapeutic options. Ann Surg Oncol 24:2827–2835. 10.1245/S10434-017-5964-6 28766218 10.1245/s10434-017-5964-6

[49] Boccardo F, Santori G, Villa G et al (2022) Long-term patency of multiple lymphatic-venous anastomoses in cancer-related lymphedema: a single center observational study. Microsurgery 42:668–676. 10.1002/MICR.30944 35916247 10.1002/micr.30944

[50] Borman P, Koyuncu EG, Yaman A et al (2021) The comparative efficacy of conventional short-stretch multilayer bandages and velcro adjustable compression wraps in active treatment phase of patients with lower limb lymphedema. Lymphat Res Biol 19:286–294. 10.1089/LRB.2020.0088 33270499 10.1089/lrb.2020.0088

[51] Karafa M, Karafova A, Szuba A (2018) The effect of different compression pressure in therapy of secondary upper extremity lymphedema in women after breast cancer surgery. Lymphology 51:21–37 (**(accessed 17 Dec 2025).**) 30248729

[52] García-Chico C, López-Ortiz S, Lorenzo-Crespo C et al (2025) Wrapping up the evidence: bandaging in breast cancer-related lymphedema-a systematic review and meta-analysis. Breast Cancer 32:654–675. 10.1007/S12282-025-01693-8 40164942 10.1007/s12282-025-01693-8

[53] Abakay H, Dogan H, Calis HT et al (2021) Is the effect of complex decongestive therapy the same for primary and secondary lower lymphedema? Lymphat Res Biol 19:165–174. 10.1089/LRB.2020.0023 32780623 10.1089/lrb.2020.0023

