# Supplementary material for: Impact of cancer-related and primary lymphedema and compression bandaging on limb range of motion: a cross-sectional study
Source: Support Care Cancer. 2026 Mar 11;34(4):306. doi: 10.1007/s00520-026-10454-y (PMC12979315; doi:10.1007/s00520-026-10454-y)
Supplement: Supplementary file 1 — (DOCX.485 KB) [file 520_2026_10454_MOESM1_ESM.docx]

***Online Resource 1:*** *Box-and-whisker plot representing the median (line within the box), the interquartile range (length of the box), the 90^th^ and the 10^th^ percentiles (whiskers above and below the box) of the maximal ROM of the ankle (A) and its two components: the extension (B) and the flexion (C) of the limb with primary (L_P) and secondary (L_S) lymphedema and the bandaged limb with primary (B_P) and secondary (B_S) lymphedema.*

*Box-and-whisker plot representing the median (line within the box), the interquartile range (length of the box), the 90^th^ and the 10^th^ percentiles (whiskers above and below the box) of the dynamic ROM of the ankle (E) and its two components: the extension (F) and the flexion (G) of the limb with primary (L_P) and secondary (L_S) lymphedema and the bandaged limb with primary (B_P) and secondary (B_S) lymphedema.*


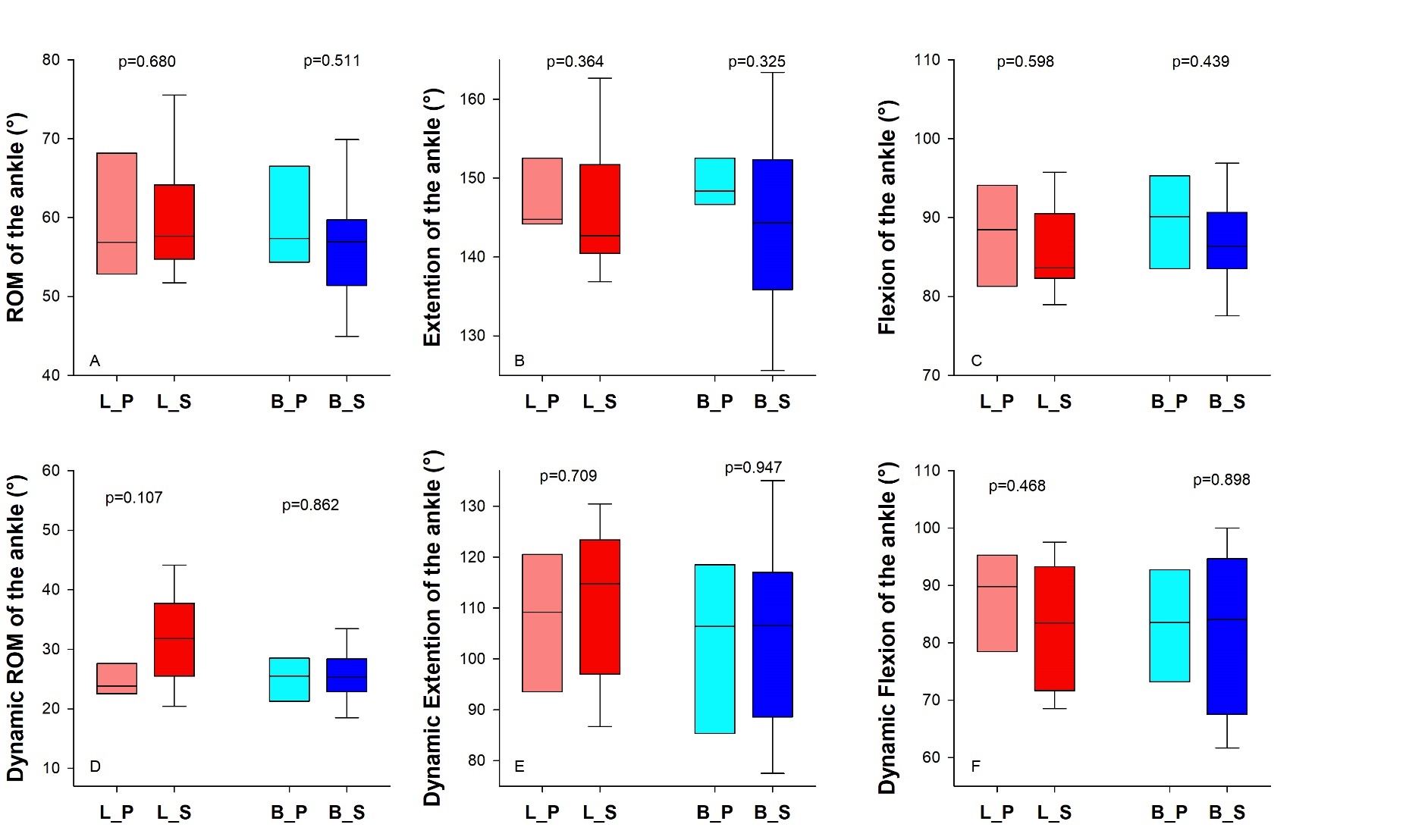


***Online Resources 2:*** *Box-and-whisker plot representing the median (line within the box), the interquartile range (length of the box), the 90^th^ and the 10^th^ percentiles (whiskers above and below the box) of the maximal ROM of the knee (A) and its two components: the extension (B) and the flexion (C) of the limb with primary (L_P) and secondary (L_S) lymphedema and the bandaged limb with primary (B_P) and secondary (B_S) lymphedema.*

*Box-and-whisker plot representing the median (line within the box), the interquartile range (length of the box), the 90^th^ and the 10^th^ percentiles (whiskers above and below the box) of the dynamic ROM of the knee (E) and its two components: the extension (F) and the flexion (G) of the limb with primary (L_P) and secondary (L_S) lymphedema and the bandaged limb with primary (B_P) and secondary (B_S) lymphedema.*

*
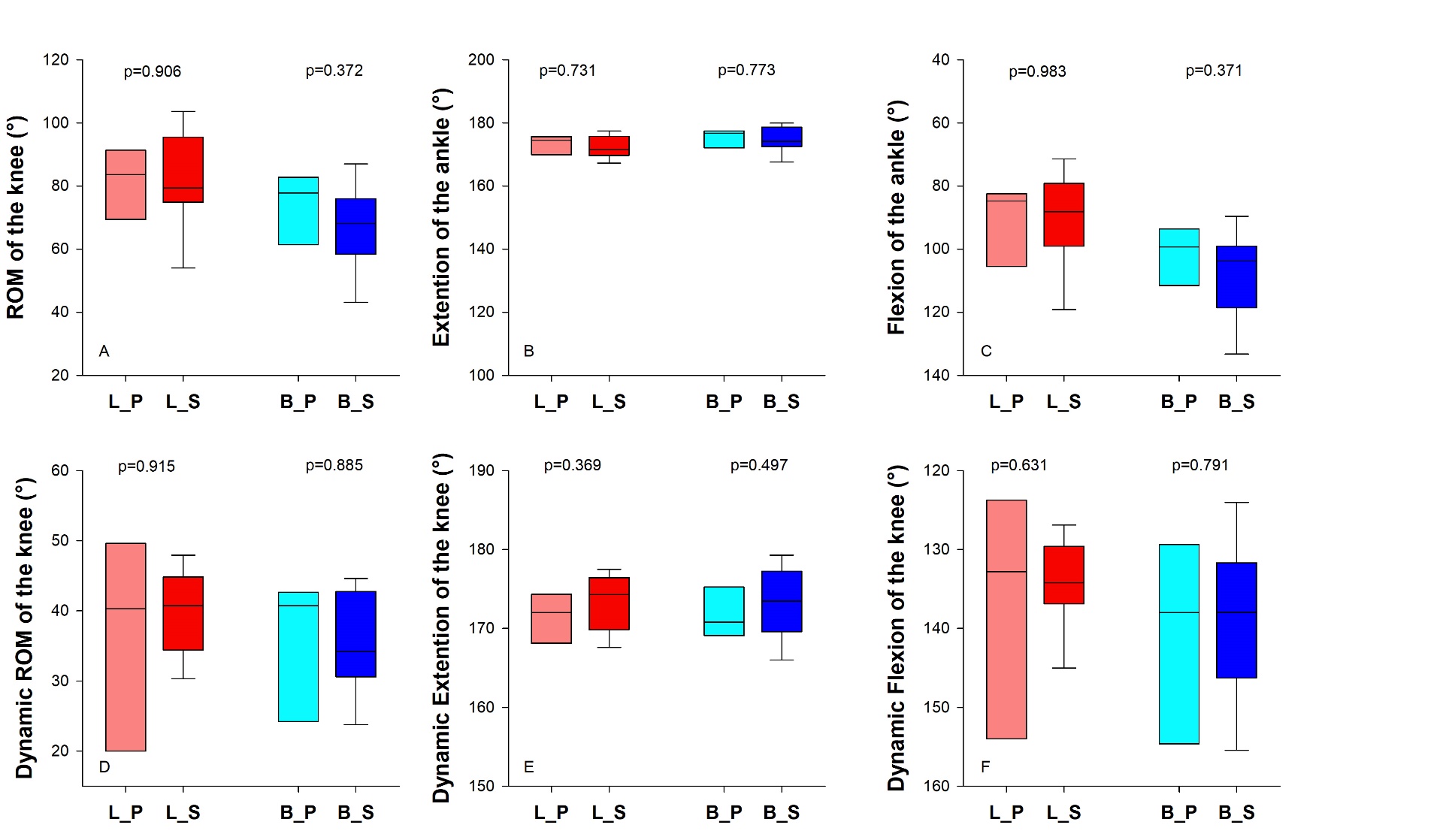
*
